# Supplementary material for: Permeability and Toxicity of Cryoprotective Agents in Silkworm Embryos: Impact on Cryopreservation
Source: Int J Mol Sci. 2024 Oct 23;25(21):11396. doi: 10.3390/ijms252111396 (PMC11546613; doi:10.3390/ijms252111396)
Supplement: Supplementary file 1 [file ijms-25-11396-s001.zip › Supplementary Materials Figure S1.pdf]

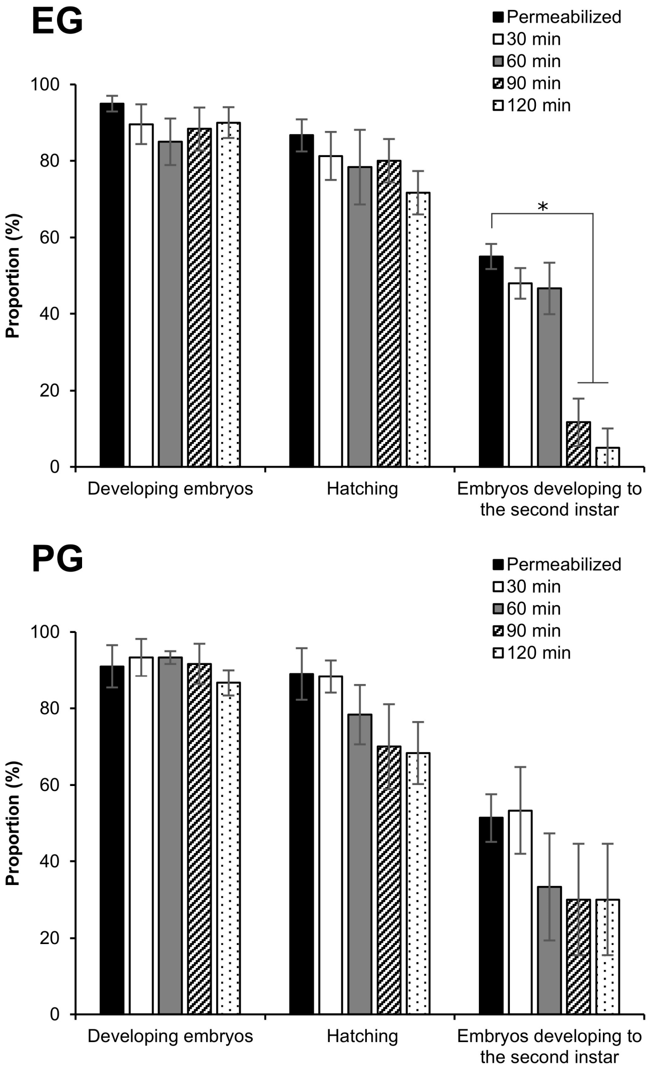

Figure S1. Effect of exposure time in 1 M EG and PG solutions in embryos at early 1 phase-stage 25 (appearance of taenidium; 160 h AEL). The permeabilized embryos of the pnd-w1 strain were exposed to 1 M EG and PG solutions for 30, 60, 90 and 120 min at 25°C. Permeabilized represents no exposure to CPAs. The experiment was repeated four to five times (n = 4-5); 10-12 embryos were used in each replicate experiment. Bars and error bars represent the mean ± SE. * $p < 0.05$ (Kruskal-Wallis test and post hoc Dunn's test).
